# Complete chloroplast genome of *Chondria tumulosa* (Ceramiales, Rhodophyta), a recently described cryptogenic species with invasive traits from Papahānaumokuākea Marine National Monument, Hawaiʻi

**DOI:** 10.1080/23802359.2021.1984327

**Published:** 2021-10-05

**Authors:** Monica O. Paiano, Randall K. Kosaki, Taylor M. Williams, Heather L. Spalding, Alison R. Sherwood

**Affiliations:** a School of Life Sciences, University of Hawaiʻi, Honolulu, HI, USA; b NOAA, Papahānaumokuākea Marine National Monument, Honolulu, HI, USA; c Department of Biology, College of Charleston, Charleston, SC, USA

**Keywords:** Manawai, Pearl and Hermes Atoll, phylogenomic analysis, Rhodomelaceae, Papahānaumokuākea

## Abstract

The complete chloroplast genome of *Chondria tumulosa*, a red alga from Manawai (Pearl and Hermes Atoll), Hawai‘i, was determined and analyzed using next-generation sequencing and *de novo* assembly approaches. The chloroplast genome sequence of *C. tumulosa* was 172,617 bp and contained 231 genes, consisting of 197 protein-coding genes, 29 transfer RNA genes, three ribosomal RNA genes, one transfer-messenger RNA gene, one non-coding RNA gene, and one intron inserted into the *trnM* gene. The number of genes and genome structure was largely similar to other members of the family Rhodomelaceae. The phylogenomic analysis of 32 complete cpDNA from the red algal order Ceramiales showed that *C. tumulosa* is a distinct species within the Chondrieae tribe, and is a diverging early relative to the other three available *Chondria* chloroplast genomes.

A recently described red alga of unknown origin was first observed in 2016 by National Oceanographic and Atmospheric Administration (NOAA) researchers at Manawai (Pearl and Hermes Atoll), Hawai‘i, forming extensive mats up to 18 cm thick, composed of distinct, entangled thalli. In August 2019, a survey reported dramatic spread of that same species, extending to 19 m depth, and surrounding the eastern, western, and northern sides of Manawai. The alga was identified as belonging to *Chondria* (Rhodomelaceae, Ceramiales). In 2020, Sherwood et al. conducted a taxonomic determination based on morphological and molecular features, and proposed a taxonomic name for the nuisance alga, *Chondria tumulosa* A. R. Sherwood and J. M. Huisman 2020. The genus *Chondria* has been reported worldwide, with 80 currently accepted species to date (Guiry and Guiry 2021).

Here, we report the determination of the complete *C. tumulosa* plastid genome by next-generation sequencing methods.

*Chondria* specimens were collected from Manawai, Papahānaumokuākea Marine National Monument, Hawai‘i, USA (27°57.9672′N, 175°46.467′W) at 19 m depth, and were preserved in silica gel for molecular analysis. A specimen was deposited at the Bernice P. Bishop Museum (www.bishopmuseum.org, Barbara Kennedy—bkennedy@bishopmuseum.org) under the accession BISH 780773. Genomic DNA was extracted using the OMEGA E.Z.N.A.^®^ Plant DNA DS Kit (OMEGA Biotek, Norcross, GA, USA) and the manufacturer’s suggested protocol. The 150 bp paired-end (PE) library construction and sequencing were performed by the GENEWIZ Corporation (South Plainfield, NJ, USA) using the Illumina MiSeq system. A total of 2.2 GB of PE sequence data was generated and used to perform *de novo* assembly in Geneious Prime (http://www.geneious.com), using the Geneious assembler. Finally, the assembled chloroplast genome was annotated using DOGMA (Wyman et al. 2004) and MFannot, and subsequently adjusted manually. Transfer RNA genes were identified using tRNAscan-SE Search Server (Schattner et al. 2005). The annotated cp genome of *C. tumulosa* is openly available in GenBank of NCBI at (https://www.ncbi.nlm.nih.gov) under the accession number MW309501. The associated BioProject, SRA, and Bio-Sample numbers are PRJNA743346, SRP327442, and SAMN20023810, respectively.

The complete chloroplast genome had a circular organization with a length of 172,617 bp, with an overall GC content of 27.4%, which is slightly lower than the average of 28.5% for the family Rhodomelaceae (Díaz-Tapia et al. 2017). The plastid genome contained 231 genes including 197 protein-coding, 29 tRNAs, three RNAs, one tmRNA, one ncRNA, and one group I intron interrupting the *trnM* gene. On the whole, the gene numbers and genome structure were very similar among species of the family Rhodomelaceae. The length of the coding region was 140,432 bp, which corresponds to 81.3% of the total length. For the phylogenomic analysis, Maximum Likelihood (ML) analysis was performed using RAxML-HPC2 on XSEDE v. 8.2.10 (Stamatakis 2006) *via* the CIPRES gateway (Miller et al. 2010) with 1000 bootstrap replicates and using the GTRCAT model. Bayesian inference was performed using the MrBayes plug-in v. 3.2.6 (Ronquist and Huelsenbeck 2003) through Geneious 2020.2.3 using four chains of Metropolis-coupled Markov Chain Monte Carlo for 1,000,000 generations and with sampling every 100 generations; 100,000 chains were removed as burn-in before determining posterior probabilities. The phylogenomic tree was constructed using previously published complete plastid genome sequences from 31 red algal species within the order Ceramiales and showed *C. tumulosa* positioned as sister to three representatives of *Chondria* identified only to the genus level, forming a sister clade to the Laurencieae tribe, with full support (Figure 1). The previous phylogenetic analysis based on the *rbcL* and SSU genes (plastid and nuclear) showed *C. tumulosa* as distinct from the other species of the genus (Sherwood et al. 2020). In the present study, phylogenomic analysis based on the complete chloroplast genome sequence reaffirms the position of *C. tumulosa* as a new species within the Chondrieae tribe of the red algal family Rhodomelaceae. Although efforts are being made to trace the origin of this red alga, it is currently classified as cryptogenic. Further analysis of additional *Chondria* species from adjacent islands in the Pacific will be necessary to elucidate the origin of *C. tumulosa* and determine whether *C. tumulosa* is an invasive species introduced from another region, or an endemic, native alga proliferating rapidly in the Papahānaumokuākea Marine National Monument.

## Data Availability

The data that support the findings of this study are available in GenBank at https://www.ncbi.nlm.nih.gov, reference number MW309501. The associated BioProject, SRA, and Bio-Sample accession numbers are PRJNA743346, SRP327442, and SAMN20023810, respectively, and can be accessed at https://www.ncbi.nlm.nih.gov/sra/?term=PRJNA743346. Molecular phylogeny of *Chondria tumulosa* and 31 other representative species of the Ceramiales (Rhodophyta). Complete chloroplast genomes were downloaded from GenBank and the phylogenomic tree was constructed by the Maximum Likelihood method with 1000 bootstrap replicates, and Bayesian Inference. Numbers along branches indicate nodal support (first value = bootstrap support, second value = Bayesian posterior probabilities). Nodes with full support are indicated with an asterisk. Scale bar = substitutions per site.
